# Groupitizing Improves Estimation of Numerosity of Auditory Sequences

**DOI:** 10.3389/fnhum.2021.687321

**Published:** 2021-06-21

**Authors:** Giovanni Anobile, Elisa Castaldi, Paula A. Maldonado Moscoso, Roberto Arrighi, David Burr

**Affiliations:** ^1^Department of Neuroscience, Psychology, Pharmacology and Child Health, University of Florence, Florence, Italy; ^2^Department of Translational Research and New Technologies in Medicine and Surgery, University of Pisa, Pisa, Italy

**Keywords:** approximate number system, groupitizing, auditory numerosity, calculation, numerosity perception, subitizing

## Abstract

Groupitizing is a recently described phenomenon of numerosity perception where clustering items of a set into smaller “subitizable” groups improves discrimination. Groupitizing is thought to be rooted on the subitizing system, with which it shares several properties: both phenomena accelerate counting and decrease estimation thresholds irrespective of stimulus format (for both simultaneous and sequential numerosity perception) and both rely on attention. As previous research on groupitizing has been almost completely limited to vision, the current study investigates whether it generalizes to other sensory modalities. Participants estimated the numerosity of a series of tones clustered either by proximity in time or by similarity in frequency. We found that compared with unstructured tone sequences, grouping lowered auditory estimation thresholds by up to 20%. The groupitizing advantage was similar across different grouping conditions, temporal proximity and tone frequency similarity. These results mirror the groupitizing effect for visual stimuli, suggesting that, like subitizing, groupitizing is an a-modal phenomenon.

## Introduction

Humans exploit various strategies to gauge the number of objects in a set, including serial counting and approximate estimation. Although estimation is relatively fast, it is prone to errors, with response variability (standard deviation of the estimates) tending to scale linearly with the number of objects (Weber Law) (Whalen et al., 1999; Ross, 2003). Interestingly, both serial counting and estimation change characteristics when the set of items is small–between 1 and 4 objects–a range known as *subitizing* (Kaufman et al., 1949). Numerosity judgements within the subitizing range violates Weber law, as people usually do not make estimation errors even when stimuli are presented for just a few milliseconds (Revkin et al., 2008; Choo and Franconeri, 2014). Similarly, serial counting response times are fast and constant within the subitizing range, then steadily increase for higher numerosities, with a clear performance discontinuity around 4 or 5 items (Kaufman et al., 1949). Subitizing was first reported by Jevons (1871), and has since been observed in numerous studies, making it one of the most robust and widely described phenomena in the numerosity literature. The subitizing phenomenon is neither restricted to arrays of items presented simultaneously over a given region of space nor to vision. Indeed, subitizing has been reported for haptic spatial arrays, and for sequences of visual, and auditory stimuli (Riggs et al., 2006; Repp, 2007; Camos and Tillmann, 2008; Gallace et al., 2008; Plaisier et al., 2009, 2010; Ferrand et al., 2010; Plaisier and Smeets, 2011; Anobile et al., 2019).

Recent studies have described a new phenomenon in numerosity perception, termed *groupitizing*, which shares many characteristics with the subitizing phenomenon. Groupitizing can be defined as “the ability to capitalize on grouping information to facilitate enumeration processes” (Starkey and McCandliss, 2014). When an array of more than ∼4 objects (above the subitizing limit) is spatially clustered into sub-groups each containing few items, with both number of groups and items per group falling within the subitizing range, the counting speed robustly increases compared with unstructured arrays (Wender and Rothkegel, 2000; Starkey and McCandliss, 2014). Signatures of grouping strategies in numerosity perception have also been observed in young chicks. Birds spontaneously prefer arrays grouped into clusters (defined by colors and shapes) containing the same number of items (Loconsole et al., 2021). Discrimination also improves when objects are presented within groups (Rugani et al., 2017).

While groupitizing has been studied much less than subitizing, the advantage in numerosity processing appears to be consistent and robust. For example, counting speed increases for objects randomly scattered over a given space but grouped by color proximity (Ciccione and Dehaene, 2020). Groupitizing also lowers perceptual thresholds (as defined by the normalized standard deviation of estimations) for approximate numerosity estimation of briefly presented stimuli (Anobile et al., 2020): clustering dot arrays into separate groups by spatial or color proximity leads up to 20% improvement in the precision of numerosity estimates. The groupitizing advantage was not restricted to spatial numerosity (items presented simultaneously) but also generalized to temporal numerosity. For example, Anobile et al. (2020) presented sequences of flashes that were all colored the same (“unstructured condition”) or grouped by color proximity (e.g., two red, two yellow, two blue). Estimation errors followed Weber’s law in both conditions, suggesting that participants did not count the items but estimated their numerosity approximatively. Most importantly, sensory precision was again improved by groupitizing up to about 15%.

Both subitizing and groupitizing share a similar reliance on attentional resources. When participants were asked to estimate the numerosity of dot arrays within the subitizing range under a condition of attentional deprivation (dual-task paradigm), the classical subitizing advantage on sensory thresholds completely vanished, with precision thresholds increasing to match those of higher numerosities (Vetter et al., 2008; Anobile et al., 2012, 2019). Similarly depriving attentional resources via a concurrent visual dual task induced significant detrimental effects on sensory thresholds for grouped arrays relatively to unstructured arrays (Maldonado Moscoso et al., 2020), suggesting that like subitizing, groupitizing relies on attentional resources.

While groupitizing has been demonstrated across different formats (spatial arrays and temporal sequences), for both counting and estimations tasks, it has mainly been studied within the visual domain. The only study (to the best of our knowledge) that has investigated the effect of stimuli grouping in another sensory modality (audition) reported increased accuracy for regular sequences of sounds organized in small equal groups (structured sequences) relative to unstructured sequences (Hoopen and Vos, 1979b). The results showed that grouping stimuli (with elements in a group not exceeding 5) improved numerical estimation accuracy, but only for short ISIs (Hoopen and Vos, 1979b). However, these results were based on error rates, an index that confounds precision and bias, and does not consider error magnitude, and are therefore difficult to relate to modern studies showing perceptual advantages of groupitizing.

The aim of the present study is to examine whether groupitizing is a general phenomenon that occurs in senses other than vision: specifically in audition. We devised an experimental paradigm in which auditory stimuli were grouped in two different ways, to mimic as much as possible grouping cues exploited in previous visual experiments. Participants estimated the numerosity of a series of tones clustered either by proximity in time (mimicking the visual grouping by spatial cues) or by similarity in frequency (mimicking the visual grouping by color). The hypothesis is straightforward: if groupitizing is at least partially rooted into the subitizing system we expect to observe lower numerosity estimation thresholds when auditory groupitizing is facilitated.

## Materials and Methods

### Participants

Fourteen adults participated in the study: four authors (GA, EC, RA, PM) and ten naïve students from the School of Psychology of Florence with little or no experience of psychophysical experiments (mean age = 29 years, standard deviation = 6 years, range = 19–45 years). The sample size was based on previous studies on groupitizing (Anobile et al., 2020; Maldonado Moscoso et al., 2020), all participants had normal or corrected-to-normal vision, and none had mathematical or other learning disorders, nor did any have substantial musical training. The research was approved by the local ethics committee (“*Commissione per l’Etica della Ricerca*,” University of Florence, July 7, 2020, n. 111) and informed consent was obtained from all participants prior to testing.

### General Procedure

Stimuli were generated and presented with PsychToolbox routines (Kleiner et al., 2007) for Matlab (ver. R2017b, The Mathworks, Inc.^1^). Participants sat 57 cm from a 15″ screen monitor (60 Hz), in a quiet and dimly light room. Stimuli were temporal sequences of 50 ms pure tones ramped on and off with 20-ms raised cosine ramps, presented with an intensity of 80 dB (at the sound source) and digitized at a sample frequency of 8192 Hz. Sounds were presented through high-quality headphones (Microsoft LifeChat LX-3000).

**FIGURE 1:**
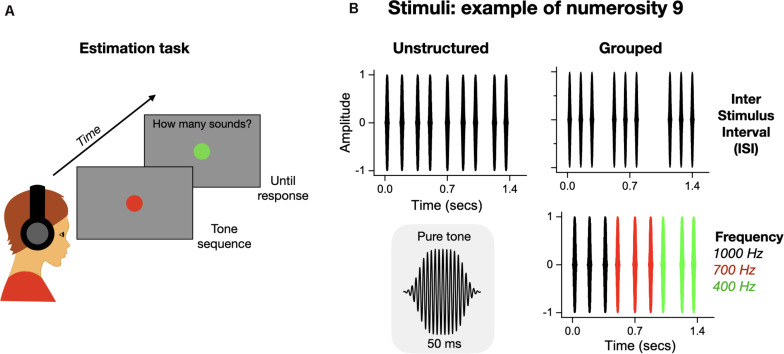
Illustration of the procedure and stimuli. **(A)** Illustration of the numerosity estimation task. Participants kept gaze on a red central fixation point while a sequence of tones was played for 1.4 s. After the auditory stimulus had finished, the fixation point color changed from red to green, signaling to verbally report the perceived numerosity. **(B)** Example of auditory stimuli for numerosity nine in the three experimental conditions: unstructured, grouped by ISI and grouped by frequency. The gray insert shows the waveform of a single pure tone.

Each trail started with the participant observing a gray blank screen on which appeared a red central fixation point (2 deg of diameter). After 200 ms, a sequence of sounds was played, with the fixation point that remained visible during the whole presentation. At the end of the auditory sequence, the fixation point turned green, to prompt the participants to provide a response (Figure 1A). On each trial, participants were asked to verbally report the number of perceived sounds, which was recorded by the experimenter via a computer keyboard. There was no time pressure on responses, but participants were asked to respond as accurately as possible. Each sequence could contain 5–12 tones, and participants were informed about the numerosity range. As the interval between the first and last sounds was always kept constant, each sequence lasted 1.4 s independently of the number of tones. As a consequence, numerosity correlates with temporal frequencies ranging from 3.5 Hz (for numerosity 5) to 8.5 Hz (for numerosity 12). As the purpose of this study was to investigate approximate numerosity estimation and not serial counting, this frequency range was chosen based on previous studies (Anobile et al., 2018, 2020) showing that in these regimes participants cannot serially count the items one-by-one, but they have to rely on approximate estimations (obeying Weber’s law).

The experiment comprised three main conditions (tested in separate sessions) in which sound sequences were manipulated to either facilitate perceptual grouping or not (details in the stimuli section). Participants (except the four authors) were not informed about the experimental conditions and were left free to choose the best strategy to solve the task. For each condition, the testing phase was preceded by a familiarization session of 22 trials (not included in the analyses). During familiarization, all numerosities were randomly presented without feedback. After the familiarization phase, the testing phase started. For each of the three experimental conditions, each participant performed around 25 trials for each numerosity (for a total of 8338 data points across all the experiments and participants). The sessions order was randomized across participants and participants had a break of ∼10 min after each session.

#### Auditory Stimuli

Participants were tested in three different conditions: (1) unstructured sequence of tones, (2) sequences grouped by tone frequency, or (3) sequences grouped by inter stimuli interval (ISI) (Figure 1B).

The unstructured sequences were built in two steps. On each trial, the whole sequence was divided into regular intervals (total duration/numerosity), with all consecutive pair of sounds demarking an identical ISI. The ISIs of these regular patterns for each numerosity were: N5 = 287 ms, N6 = 220 ms, N7 = 175 ms, N8 = 142 ms, N9 = 118 ms, N10 = 100 ms, N11 = 85 ms; N12 = 72 ms (average = 150 ms, SD = 73 ms). Then to reduce the regularity of the tone sequences, a small temporal jitter (around 10% of the ISI for regular patterns of that numerosity) was applied to the timing of each tone (excluding the first and the last), by increasing or reducing the ISI between two consecutive impulses (sign of the perturbation randomly selected trial by trial for each tone). On any given trial, all tones were defined by an identical frequency randomly selected out of three possibilities: 400, 700, or 1000 Hz.

The temporal structures of the sequences grouped by tone frequency were identical to those used for the unstructured stimuli, except for the frequency of the tones in the sequence: the tones were divided into groups of impulses of identical frequency. Each group of tones had frequencies of 400, 700, or 1000 Hz. The sequence clustering followed the groupitizing rules: the total sequence was divided into two or three groups, each containing two to four tones (see Figure 1B for an example of numerosity nine clustered into three groups of three tones each). Each numerosity yielded a given number of possible clusters: N5 (2 + 3 or 3 + 2), N6 (3 + 3 or 2 + 2 + 2), N7 (3 + 2 + 2 or 2 + 2 + 3 or 3 + 4), N8 (4 + 4 or 2 + 2 + 2 + 2), N9 (4 + 3 + 2 or 3 + 3 + 3), N10 (4 + 4 + 2 or 3 + 4 + 3), N11 (4 + 4 + 3), N12 (4 + 4 + 4). On every trial, for the selected numerosity, we randomly selected one of the possible patterns (e.g., for N = 8 the choice was between four groups of two tones or two groups of four tones). Finally, to limit the possibility of solving the task by simply memorizing the correspondence between a given numerosity and a sequence of sound frequencies, we arbitrarily defined up to six different frequency configurations for each numerosity. For example, on each trial in which numerosity “six” was presented, the frequency of the sounds in the sequence was defined by one of the following pattern: [1,000, 10,00, 700, 700, 400, 400] or [1,000, 1,000, 400, 400, 700, 700] or [700, 700, 1000, 1000, 400, 400] or [700, 700, 700, 400, 400, 400] or [400, 400, 400, 1,000, 1,000, 1,000], or [1,000, 1,000, 1,000, 700, 700, 700] Hz.

The sequences grouped by inter-stimulus interval (ISI) were also built in two steps. First the whole sequence (1.4 s) was divided into 12 identical intervals (with 12 corresponding to the highest numerosity tested). Then some of the slots were selected to create temporally separate tone clusters (see Figure 1B for an example of numerosity nine clustered into three groups of three tones each). In this condition, we did not apply any temporal jitter to the sequences. The tone clusters were created according to the groupitizing rules: 2, 3, or 4 groups each containing few items (from 1 to 5). The only exception was the numerosity eleven that was created by a group of 5 and a group of 6 tones. The ISI between groups ranged between 140 to 942 ms. To keep the conditions balanced, the numerosity12 was played but as no clustering could have been applied (all slots in the sequence were used), this numerosity was eliminated from the analyses. The temporal clusters were: N5 (2 + 3 or 2 + 1 + 2), N6 (3 + 3 or 2 + 2 + 2), N7 (2 + 3 + 2 or 3 + 1 + 3), N8 (4 + 4 or 2 + 2 + 2 + 2), N9 (2 + 3 + 4 or 3 + 3 + 3), N10 (5 + 5 or 4 + 2 + 4), N11 (5 + 6), N12 (no clusters). On every trial, for each numerosity, we randomly selected one of the two possible patterns (e.g., for N8 four groups of two tones or two groups of four tones). On each trial, all the tones in the sequence were defined by three possible frequencies: 400, 700, or 1,000 Hz.

### Data Analyses

We first checked for response outliers. Separately for each participant, condition and numerosity, we eliminated trials below or above 3 SD of accuracy or response time (∼2% of the trials for each condition for a total of 190 trials). Given that in the ISI condition only numerosities from 5 to 11 provided clustering cues, numerosity twelve was not included in the analyses. For each participant, numerosity and condition we separately calculated the average perceived numerosity and the standard deviation of the responses. Sensory precision was measured by normalizing the standard deviation by the physical numerosity to obtain a Coefficient of variation (CV), a dimensionless index of precision that allows comparison and averaging of performance across different numerosities.

(1) $$C\,V=\frac{\sigma }{N}$$

where *N* is numerosity and σ standard deviation of responses to that numerosity. The percentage of advantage of the CVs in the grouping compared with unstructured condition was indexed as the percent improvement:

(2) $$Groupingadvantage\left(\%\right)=\frac{C\,V\,u-C\,V\,g}{C\,V\,u}\times 100$$

Where *CV*_*u*_ and *CV*_*g*_ are the Coefficients of variation for the unstructured and grouped conditions, respectively.

Data were analyzed by repeated measures ANOVA (3 conditions × 7 numerosities) and *post-hoc t*-tests. *P*-values (two-tailed) were corrected for multiple comparisons with the Bonferroni method (p_bonf_). Effect sizes associated with ANOVA were reported as η^2^, and those associated with *post-hoc t*-tests as Cohen’s d. *T*-tests were supplemented with Bayesian statistics, calculating Bayes Factors, the ratio of the likelihood of the alternative to the null hypothesis, and reporting them as base 10 logarithms. By convention, LogBF > 0.5 is considered substantial evidence in favor of the alternative hypothesis and LogBF < −0.5 substantial evidence for the null hypothesis. Absolute values greater than 1 are considered strong evidence, and greater than 2 definitive. Data were analyses using JASP (ver. 0.8.6 2018) and Matlab (ver. R2017b) software.

## Results

### Effect of Auditory Groupitizing on Perceived Numerosity

**FIGURE 2:**
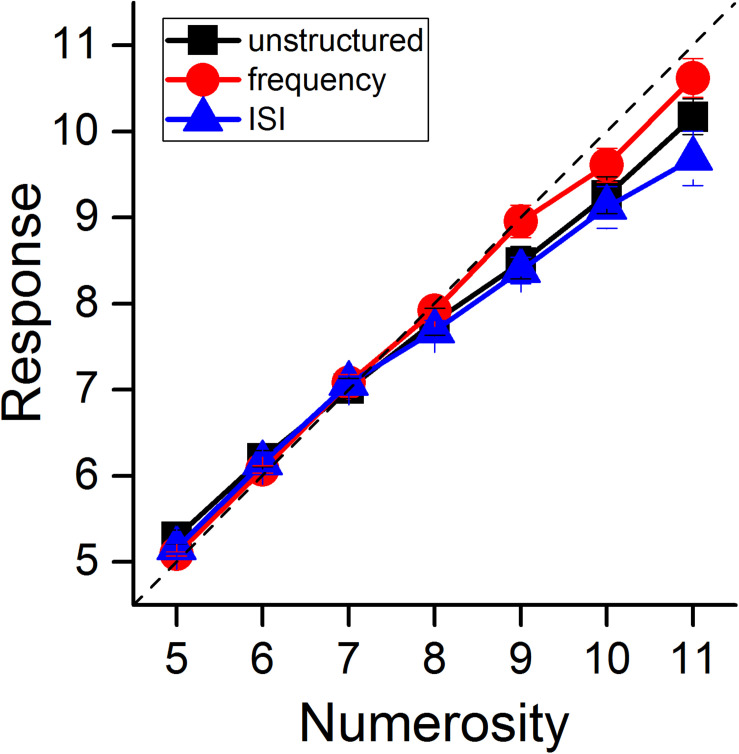
Perceived auditory numerosity. Average perceived numerosity as a function of physical numerosity for the three experimental conditions (black squares: unstructured stimuli, red circles: stimuli grouped by frequency, blue triangles: stimuli grouped by ISI). Error bars are ± 1 SEM.

We first evaluated the effect of grouping on perceived numerosity. Figure 2 shows average responses separately for the three experimental conditions, as a function of physical numerosity. To statistically test differences across conditions, we performed a repeated measures ANOVA with numerosity (7 levels, from N5 to N11) and grouping condition (3 levels) as within subject factors. The main effect of numerosity was obviously significant [*F*(6, 78) = 445, *p* < 0.001, η^2^ = 0.97], but there was no significant effect of “grouping condition” [*F*(2, 26) = 2.09, *p* = 0.14, η^2^ = 0.14]. The condition-by-numerosity interaction was statistically significant [*F*(12, 156) = 4.73, *p* < 0.001, η^2^ = 0.26]. To explore this interaction, we performed a series of *post-hoc t*-tests contrasting, for each numerosity, the responses in the unstructured condition against those for grouping by ISI or frequency separately. None of the numerosity estimates in the grouping conditions significantly differed from the unstructured condition after Bonferroni correction (min p_bonf_ = 0.11 for the contrast N5 unstructured Vs. N5 frequency, all the other p_bonf_ > 0.42; highestLogBF = 0.84 for the same comparison, all the other –0.54 < LogBF < 0.33). Overall, these results indicate that auditory grouping had no strong effect on average perceived numerosity of auditory stimuli.

### Effect of Auditory Groupitizing on Sensory Precision

**FIGURE 3:**
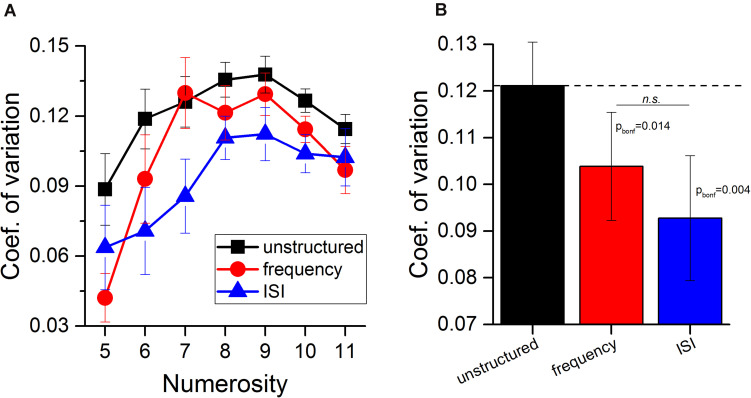
Groupitizing affects precision of estimation of auditory stimuli. **(A)** Average Coefficient of variation as a function of numerosity for the three experimental conditions (black squares: unstructured stimuli, red circles: stimuli grouped by frequency, blue triangles: stimuli grouped by ISI). **(B)** Coefficients of variation averaged across numerosity levels and participants. Black Error bars show ± 1 SEM.

Having verified that average perceived numerosity did not change with grouping, we focused on sensory precision, indexed by the Coefficient of variation (standard deviations normalized by numerosity; see Materials and Methods). Figure 3A shows the average Coefficient of variation as a function of numerosity, for all three experimental conditions. It is evident on inspection that unstructured stimuli (black squares) yielded higher Coefficients of variations (less precision) than the two grouping conditions. Figure 3B shows the Coefficient of variation averaged across numerosities and participants for the unstructured (black) and the two grouping conditions (red: frequency, blue: ISI).

Repeated measure ANOVA with numerosity (7 levels, from N5 to N11) and grouping condition (3 levels) as within subject factors revealed a main effect of condition [*F*(2, 26) = 7.83, *p* = 0.002, η^2^ = 0.38]. The factor numerosity was also statistically significant [*F*(6, 78) = 8.6, *p* < 0.001, η^2^ = 0.40], while the condition-by-numerosity interaction was not [*F*(12, 156) = 1.76, *p* = 0.06, η^2^ = 0.12]. *Post hoc t*-tests on conditions revealed that both grouping by frequency (*t* = 3.4, p_bonf_ = 0.014, Cohen’s *d* = 0.9, LogBF = 2.19) and by ISI (*t* = 4.1, p_bonf_ = 0.004, Cohen’s *d* = 1.1, LogBF = 4.9) significantly improved sensory precision compared to the unstructured condition. The two grouping conditions did not differ between each other (*t* = 1.2, p_bonf_ = 0.7, Cohen’s *d* = 0.32, LogBF = –0.52).

Although the condition-by-numerosity interaction in the ANOVA was not statistically significant, to test whether different strategies (such as counting) may have been used to solve at high and low numerosities, we further investigated the dependence on numerosity by dividing the data into high and low numerosities (greater or less than 7.5). The improvement with groupitizing was strong and significant for both ranges [N5–7: mean = 25%, t_(__83)_ = 4.8, *p* < 0.001, Cohen’s *d* = 0.53, LogBF = 3.2; N9–11: mean = 15%, t_(__83)_ = 3.59, *p* < 0.001, Cohen’s *d* = 0.39, LogBF = 1.6]. The size of the effect was statistically indistinguishable in the two numerical ranges [*t*_(__83)_ = 1.58, *p* = 0.12, Cohen’s *d* = 0.17, LogBF = –0.40].

**FIGURE 4:**
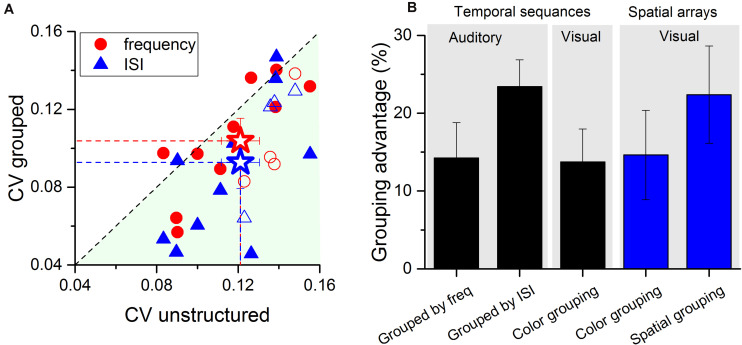
Individual coefficients of variation for the three conditions. **(A)** Scatter plot of Coefficient of variation (CV) in the grouped conditions (red circles: stimuli grouped by frequency, blue triangles: stimuli grouped by ISI) plotted against those measured in the unstructured condition. For the grouping by ISI, the average CV was 0.09 (blue star and dashed line), for the grouping by frequency was 0.10 (red star and dashed line), both lower than the average CV in the unstructured condition (0.12). For almost all participants (naïve filled circles and triangles, authors open circles and triangles) CVs for grouped stimuli were lower than those for unstructured stimuli. Error bars are ± 1 SEM. **(B)** Groupitizing advantage on sensory precision across stimuli formats and sensory modalities. The first two bars report the grouping advantage for auditory stimuli (current study) grouped by frequency or by ISI (compared with unstructured stimuli). The other data show results from a previous study investigating groupitizing effects in vision (Anobile et al., 2020). Data are publicly available at Anobile et al. (2020). From left to right: grouping temporal sequences by color; grouping spatial arrays by color; grouping spatial arrays by spatial proximity. Error bars show ± 1 SEM.

Figure 4A shows the Coefficient of variation (CV) averaged across all numerosities for all participants, plotting CV measured in the two grouped conditions (frequency in red and ISI in blue) against that for the unstructured condition. Despite large inter-individual variability in thresholds, and in the improvement induced by grouping, the majority of data points fall below the equality line, indicating that most of the participants (with no obvious differences between naïve and authors, see filled and empty small data points in Figure 4A) estimated numerosity of the grouped stimuli with higher precision than the ungrouped. On average, grouping the stimuli by ISI improved precision by about 25% and grouping by frequency by 15% (with improvement defined by eqn. 2). These robust effects nicely compliment with those previously reported in vision for both, temporal sequences, or spatial arrays (improvement of about 15 and 20%, respectively) as shown by Figure 4B. An ANOVA on the grouping advantage across stimuli formats and grouping strategy revealed that the effects were statistically indistinguishable [*F*(4, 75) = 0.88, *p* = 0.47].

## Discussion

The aim of this study was to investigate whether and to what extent groupitizing effects occur in audition. The results revealed that auditory grouping cues had no measurable effect on average perceived numerosity, but they decreased estimation thresholds by up to 20%, similar to the advantage previously reported for spatial arrays. The groupitizing advantage occurred for both grouping conditions, both when groups were defined by manipulating the temporal proximity of the tone, as well as when they were defined by similarity of tone frequency.

These results mirror what has been recently reported in the visual domain, both for arrays of stimuli presented simultaneously over a given region of space and for sequences of flashes (Anobile et al., 2020), suggesting that groupitizing may reflect the activity of one or more a-modal and cross-format systems. Most evidence suggests that groupitizing depends on subitizing, an attention-dependent mechanism for fast and accurate enumeration of small quantities, combined with arithmetical strategies. Participants probably parse the array into subitizable samples, which can be precisely enumerated by leveraging on the subitizing precision, and summed together to estimate total numerosity. In support to this hypothesis, Starkey and McCandliss (2014) showed that children with higher arithmetical abilities took greater advantage from groupitizing in a dot counting task. Ciccione and Dehaene (2020) further generalized these results to the adult population by showing a stronger groupitizing advantage for mathematics university students compared with humanities and psychology. And arithmetical abilities in adults are better correlated with numerosity thresholds for grouped than ungrouped stimuli (Maldonado Moscoso et al., 2020).

(Hoopen and Vos, 1979a,b) looked at the effect of grouping of tones on perception, initially to study attentional switching. They found that at some ISIs, grouping caused underestimation of numerosity (Hoopen and Vos, 1979b), which we did not observe here. However, at ISIs compatible with those of this study, they also reported no underestimation in numerosity. They further found that for short ISIs, where counting was not possible, accuracy improved in the grouped condition. Although differences in the experimental procedures (such as using regular rather than randomized ISIs and that their measure of accuracy confounds bias and precision) make it difficult to relate in detail their study with the current study, their findings are broadly consistent with those reported here.

In our study the interstimulus intervals were generally short, making it difficult to count the stimuli: on debriefing, all participants reported that they guessed at the numerosity, and did not attempt to count them (although this was not expressly forbidden). If counting were possible, it would have occurred for the lower rather than the higher numerosities, as total stimulus duration was constant (1.4 s), and ISI varied accordingly, from 287 ms for *N* = 5–85 ms for *N* = 11. However, when we separated the data into high and low numerosities (greater or less than 7.5), we found that both ranges showed strong and highly significant groupitizing effects, with no significant difference between the two ranges. We therefore conclude that the results are unlikely to reflect counting strategies.

Over the last few years there has been increasing interest in the association between numerosity perception and mathematics. A considerable body of evidence suggests that numerosity perception may represent an early non-symbolic foundational capacity for the development of symbolic arithmetic skills (Halberda et al., 2008; Piazza, 2010; Chen and Li, 2014; Fazio et al., 2014; Schneider et al., 2017). However, despite much evidence supporting this fascinating idea, many studies have failed to find significant correlations, or causal training effects between numerosity and arithmetic (De Smedt et al., 2013; Sasanguie et al., 2014; Caviola et al., 2020; Bugden et al., 2021). The literature on this topic is contradictory, and the reasons for reported failures in correlations between numerosity perception and arithmetic are still largely unclear and debated.

One possibility is that groupitizing is the link between numerosity perception and math: people with strong arithmetic skills may take advantage of natural clustering in random arrays and use a combination of grouping and arithmetical strategies to solve the numerosity task. This in turn could drive (even partially) the correlation with math scores. A recent study found that visual and auditory subitizing capacities do not correlate with mental calculation abilities (Anobile et al., 2019). Similarly, numerosity discrimination thresholds for very high numerosity do not correlate with arithmetical abilities (Anobile et al., 2016). The fact that arithmetical abilities correlate only with intermediate numerosities (Burr et al., 2017) might be because these numerosities are ideal for groupitizing. Numerosities within the subitizing range are (by definition) immediately and accurately perceived holistically, with no need to apply arithmetic strategies to combine different subsets. On the other hand, very high numerosities might be difficult to segment and cluster into a small (subitizable) number of subgroups. Furthermore, numerosity discrimination thresholds in the estimation range predict arithmetical abilities in primary school children for spatial arrays (dots), but not for auditory or visual sequences (Anobile et al., 2018). This could reflect lower natural clustering for temporal sequences compared with spatial arrays, or the existence of multiple systems with different relationships with the development of formal arithmetic. Future research should investigate whether auditory groupitizing relates to arithmetical abilities to the same extent as visuo-spatial groupitizing does.

Clinical research may also contribute to clarifying whether the link between numerosity perception and arithmetical skills is mediated by groupitizing. Groupitizing could be studied in developmental dyscalculia, and the effectiveness of training programs promoting the use of groupitizing strategies (inducing mental arithmetical procedures), rather than generally boosting numerosity discrimination *per sè*, should be quantitatively evaluated.

Subitizing limits for auditory sequences are thought to be lower than those for spatial vision (Repp, 2007; Anobile et al., 2019), possibly because the stimuli are one-dimensional rather than two-dimensional. Indeed some studies suggest that the limit could be as low as three (Repp, 2007), while the visual limit is usually considered to be four (Jevons, 1871; Kaufman et al., 1949; Atkinson et al., 1976). However, there is no sharp cutoff for subitizing, and the limit depends somewhat on definition. For example, Piazza et al. (2011) define the limit operationally by fitting estimation errors with a Gaussian error function, and taking the 50% point as the numerosity limit. With this definition, the visual limit for spatial subitizing is around six while the auditory sequential limit is five (Anobile et al., 2019). So, while the limit for auditory sequences is probably slightly less than that for visual sequences, it is likely that the participants were able to subitize reasonably well even the longest clusters of four items. This is supported by the fact that the average groupitizing effects for auditory sequences were very comparable with those previously reported for spatial arrays (∼20% see Figure 4B).

In addition to the average values being different, the limits for spatial and temporal subitizing do not correlate with each other, suggesting separate systems (Anobile et al., 2019). It is therefore possible that auditory and spatial visual groupitizing are subserved by different mechanisms, but this issue would need to be specifically addressed in future studies. Research is also needed to explore the brain networks underlying groupitizing and how they relate to those supporting subitizing (Piazza et al., 2002; Ansari et al., 2007; He et al., 2014) and arithmetic calculation (Castaldi et al., 2020).

Counting or estimating the number of visual objects may appear a very simple and basic task compared to many other human capacities. However, the strategies used to solve these tasks may be much more complex and informative than previously thought. Groupitizing, as subitizing, is an example of this complexity and informative power.

## Data Availability Statement

The datasets presented in this study can be found in online repositories. The names of the repository/repositories and accession number(s) can be found below: Zenodo (http://doi.org/10.5281/zenodo.4638767).

## Ethics Statement

The studies involving human participants were reviewed and approved by the Commissione per l’Etica della Ricerca, University of Florence, July 7, 2020, n. 111. The patients/participants provided their written informed consent to participate in this study.

## Author Contributions

GA, EC, and PM performed the testing and data collection. GA performed the data analysis. All authors contributed to the study concept, experimental design, interpretation of results, manuscript preparation, and approved the final version of the manuscript.

## Conflict of Interest

The authors declare that the research was conducted in the absence of any commercial or financial relationships that could be construed as a potential conflict of interest.

## Footnotes

1 http://mathworks.com
